# A panel of kallikrein markers can reduce unnecessary biopsy for prostate cancer: data from the European Randomized Study of Prostate Cancer Screening in Göteborg, Sweden

**DOI:** 10.1186/1741-7015-6-19

**Published:** 2008-07-08

**Authors:** Andrew J Vickers, Angel M Cronin, Gunnar Aus, Carl-Gustav Pihl, Charlotte Becker, Kim Pettersson, Peter T Scardino, Jonas Hugosson, Hans Lilja

**Affiliations:** 1 Memorial Sloan-Kettering Cancer Center, Department of Epidemiology and Biostatistics, East 63rd Street, New York, NY 10021, USA; 2 Sahlgrenska University Hospital, Gothenburg, Sweden; 3 University Hospital UMAS, Malmö, Sweden; 4 Department of Biotechnology, University of Turku, Turku, Finland

## Abstract

**Background:**

Prostate-specific antigen (PSA) is widely used to detect prostate cancer. The low positive predictive value of elevated PSA results in large numbers of unnecessary prostate biopsies. We set out to determine whether a multivariable model including four kallikrein forms (total, free, and intact PSA, and human kallikrein 2 (hK2)) could predict prostate biopsy outcome in previously unscreened men with elevated total PSA.

**Methods:**

The study cohort comprised 740 men in Göteborg, Sweden, undergoing biopsy during the first round of the European Randomized study of Screening for Prostate Cancer. We calculated the area-under-the-curve (AUC) for predicting prostate cancer at biopsy. AUCs for a model including age and PSA (the 'laboratory' model) and age, PSA and digital rectal exam (the 'clinical' model) were compared with those for models that also included additional kallikreins.

**Results:**

Addition of free and intact PSA and hK2 improved AUC from 0.68 to 0.83 and from 0.72 to 0.84, for the laboratory and clinical models respectively. Using a 20% risk of prostate cancer as the threshold for biopsy would have reduced the number of biopsies by 424 (57%) and missed only 31 out of 152 low-grade and 3 out of 40 high-grade cancers.

**Conclusion:**

Multiple kallikrein forms measured in blood can predict the result of biopsy in previously unscreened men with elevated PSA. A multivariable model can determine which men should be advised to undergo biopsy and which might be advised to continue screening, but defer biopsy until there was stronger evidence of malignancy.

## Background

Prostate specific antigen (PSA) is the only molecular marker routinely used for the early detection of a common cancer. Nonetheless, it remains an imperfect test: although PSA is highly specific to the prostate gland, elevated blood PSA is not specific to cancer as it can be a result of benign conditions such as benign prostatic hypertrophy and prostatitis [1,2]. Accordingly, most men with elevated PSA do not have prostate cancer, with the result that many undergo prostate biopsy unnecessarily. It has been estimated that approximately 1 million biopsies are conducted per year in the USA [3]. As the annual incidence of prostate cancer is 235,000 cases [4], this suggests that, each year, over 750,000 American men are needlessly subjected to prostate biopsy, with attendant pain, inconvenience, financial costs, and risk of infection.

Analyzing data from the Prostate Cancer Prevention Trial (which was unique in biopsying men with PSAs below 1 ng/ml), Thompson and colleagues reported that no PSA cutoff was associated with good test characteristics: for cutoffs up to 4 ng/ml, sensitivity and specificity ranged, respectively, from 21% to 83% and 39% to 94%; the positive predictive value varied from 7% to 27% (see [5]). The modest diagnostic accuracy of PSA testing has led investigators to evaluate additional markers, such as ratio of free-to-total PSA [6,7], single-chain ('intact PSA') versus multi-chain ('nicked PSA') forms of PSA [8], proPSA [9-11], and human kallikrein 2 (hK2) [12-14]. Studies have generally, although not unequivocally, found that these biomarkers aid in cancer detection [7,10,14-17]. We have identified four key criteria for a high quality study of markers for prostate cancer detection. Firstly, the PSA screening history of participants must be well defined. This is because the properties of tests for prostate cancer likely change with repeat screens: prevalent cases of advanced cancer will largely be identified in early screening rounds, whereas later rounds will identify predominately incident cases; accordingly, the positive predictive value of the screening test may decrease from earlier to later rounds. Secondly, decision analytic methods should be used to analyze the results. The incremental value of markers additional to PSA is generally assessed by comparing the accuracy of a statistical model including only PSA with a model including PSA plus the additional marker. Typical metrics include positive predictive value, sensitivity, specificity or, as a global statistic of predictive accuracy, the area under the receiver operating characteristic curve (AUC). However, it is quite possible for an additional marker to improve accuracy but for this to make little practical difference in the clinic. Thirdly, kallikreins are subject to degradation in frozen, stored and thawed samples [18,19]; accordingly, measurement of fresh samples is optimal. Fourthly, the result of the study must be a readily usable statistical tool that gives the probability of a positive biopsy.

In this paper, we analyze data from the Göteborg cohort of the European Randomized study of Screening for Prostate Cancer screening (ERSPC). Our aim is to determine whether a multivariable model including four kallikrein markers (total PSA, free PSA, intact PSA and hK2) can predict the results of a prostate biopsy in previously unscreened men with elevated PSA. Prostate cancer screening has not been recommended by the Swedish national health system, and population-based research suggests that very few Swedish men underwent PSA screening at the time of this trial [20,21]. Levels of molecular markers were assessed using research assays, and decision analytic methods were applied to estimate the clinical value of the four-marker set.

## Methods

The design of the Swedish arm of the ERSPC in Göteborg, Sweden, has been previously reported [22]. In brief, the study population consisted of all males living in Göteborg, Sweden, on 12 December 1994, and who were born between 1 January 1930 and 12 December 1944 (*n *= 32,298). Of these men, 9972 were randomly selected to undergo initial PSA testing between 1995 and 1996. All men were re-invited for PSA testing every second year up to 2005, unless they were diagnosed with prostate cancer, were aged over 70 or had died. Men with a level of total PSA in serum ≥3.0 ng/ml were invited to undergo clinical examination by an experienced urologist. This examination included digital rectal examination (DRE), and transrectal ultrasound guided laterally directed sextant biopsy of the prostate [23]. All biopsy specimens were evaluated by a single pathologist. Tumors were classified according to the 1997 International Union Against Cancer staging system [24] and graded according to the Gleason grading system [25].

Seven milliliters of blood was collected by venipuncture in Vacutainer^® ^tubes from every man who signed informed consent to undergo PSA testing. The blood was allowed to clot, and serum was separated from blood cells by centrifugation at 3000 *g *for 20 minutes, separated and frozen within 3 hours from collection, and kept frozen at -20°C until analysis. Free and total PSA were measured within 2 weeks from the blood draw by Dr Lilja's laboratory at the Wallenberg Research Laboratories, Department of Laboratory Medicine, Lund University, University Hospital UMAS in Malmö, Sweden, using the dual-label DELFIA Prostatus^® ^total/free PSA-Assay (Perkin-Elmer, Turku, Finland) as reported previously [22]. During 2005 and 2006, analyses of intact PSA and hK2 were performed at Dr Lilja's laboratory. Samples had been frozen at -20°C for up to 2 years, thawed and aliquoted once, and then stored frozen at -70°C until analysis. All analyses were conducted blind to biopsy result.

The performance of the Prostatus^® ^assay has been comprehensively documented previously [26]. The combination H117/H50 detects free PSA and PSA bound in complex to 1-anti-chymotrypsin (ACT) in an equimolar fashion [27], and also fully cross-reacts with hK2 [28]. The combination of Mabs (monoclonal antibody) H117/5A10 used to measure free PSA does not cross-react with hK2 [28], or with PSA-ACT (<0.2%) [27].

Immunodetection of hK2 is based on previously reported in-house research assays [28,29] where important modifications provide enhanced low-end precision and linearity [30]. Mab (6H10) has 5% cross-reaction to PSA, but this cross-reaction to PSA is eliminated (0.005%) by the addition of PSA-blocking Mabs (2E9, 2H11, 10, 36). We have previously described the performance of this assay [31]. Our method for assaying intact PSA has been published [8,17,32]. Briefly, biotinylated 5A10 antibody is used to capture the free PSA; after incubation and a wash step, we add as detection antibody europium-labeled intact PSA antibody 5C3 that loses binding to PSA when PSA is internally cleaved at Lys145–Lys146. The analytical detection limit of the assay is 0.035 ng/ml.

### Statistical methods

#### Patients and outcomes

Data were obtained under a waiver approved by the ethical committee at Göteborg University. The sample includes only men who were biopsied. As biopsy was recommended only for men with total PSA of at least 3.00 ng/ml, our findings are applicable only to men with an elevated total PSA. We performed analyses for the outcome of a positive biopsy (yes or no) in the first screening round ('round 1'), defined as the first time that the man participated in the study irrespective of calendar year. Of note, this is a different definition from previous papers examining men in the Göteborg cohort, which required participation to take place in 1995 to 1996.

We were interested only in biopsies that occurred on the basis of screening, and not any that occurred due to external findings, such as a DRE during an off-study history and physical. Since we did not know the reason for a biopsy, only when the biopsy occurred, we defined a biopsy to be triggered by screening if it occurred within 6 months of the blood draw. High-grade cancer was defined as biopsy Gleason score 7 or higher.

#### Statistical modeling

Our overall question concerned the additional value of the new markers (free PSA, intact PSA, hK2) in two settings: a laboratory sending blood results to a doctor, and a clinical consultation between a patient and a doctor, during which a decision would be made as to whether biopsy was advisable. Accordingly, the base model for the laboratory setting therefore included age and total PSA; for the clinical setting, the base model also included the DRE result. We added the new markers (free PSA, intact PSA, hK2) one at a time to the base models to estimate the increment in predictive accuracy associated with each additional marker. We pre-specified that the full model would include the base model plus all three of the additional markers: no variable selection was employed. All markers were entered as restricted cubic splines with knots at the tertiles to allow a non-linear relationship with outcome. Multivariable logistic regression was used to fit all models. Owing to the inclusion of non-linear terms, we used a nomogram to illustrate the full model; we only included patients with total PSA of 15 ng/ml or less for the nomogram as there were very few patients with results above this value.

#### Model evaluation

Predictive accuracy was assessed by the AUC. We corrected for overfit by repeated 10-fold cross-validation. Confidence intervals and p values were obtained by bootstrapping. Since not all patients with an elevated PSA underwent a biopsy in round 1, we explored the potential effects of verification bias [33,34].

We used decision curve analysis [35] to explore the clinical effects of our models. This method estimates a 'net benefit' for prediction models by summing the benefits (true positives) and subtracting the harms (false positives). As the value of a true positive (such as finding a cancer early) may be different from the disadvantages stemming from a false positive (an unnecessary biopsy), the net benefit calculation weights true and false positives differently. The weighting is derived from the threshold probability of a disease at which a patient would opt for intervention; in the current case, the probability of prostate cancer at which a patient would choose to be biopsied: a false positive is weighted as *p*_t_/(1 - *p*_t_) compared with a true positive where *p*_t _is the threshold probability. As this threshold probability can vary from patient to patient, net benefit is calculated across a range of probabilities. We chose 10% to 40% as our range: we would be surprised if any man would choose a biopsy if told that his probability of prostate cancer is less than 10% (and few clinicians would do 10 or more biopsies to find a single cancer); similarly, it would be rare for a man to demand at least a 50:50 chance of cancer before they would accept biopsy. The interpretation of a decision curve is very simple: the model with the highest net benefit at a particular threshold probability should be chosen. Correction for overfit was by repeated 10-fold cross-validation, as detailed above, although instead of estimating the AUC, we estimated the net benefit at each threshold probability. All analyses were conducted using Stata 9.2 and R [36] with the *Design *library added [37].

## Results

### Characteristics of the cohort

In total, 7454 men participated (75% of the total of 9972 randomized to PSA screening); the year of initial participation was from 1995 to 1996 for 5855 men (79%), from 1997 to 1998 for 575 men (8%), and from 1999 to 2005 for 1024 men (14%). During their first round of participation, 956 (13%) men had elevated PSA, 858 (12%) were biopsied, and 209 (2.8%) were diagnosed with prostate cancer. We excluded 118 men from our analysis due to insufficient blood for measurement (*n *= 102) or because of unknown DRE results (*n *= 16). Our final cohort for analysis contained 740 (10%) men who were biopsied during round 1; at that time, 192 (2.6%) were diagnosed with prostate cancer.

Clinical characteristics of men biopsied in round 1 are summarized in Table 1. Participants diagnosed with cancer had higher levels of total PSA and hK2 compared with men who were not diagnosed with cancer. Median levels of free PSA and intact PSA were similar between men with and without cancer. Among those diagnosed with cancer, DRE results were abnormal in 40% and biopsy Gleason score was 7 or higher in 40 men (21%). The number of cancers diagnosed by total PSA level is shown in Table 2. As expected, as PSA increases, so does the proportion of total and high-grade cancers.

**Table 1:** Characteristics of the study cohort

	**No cancer detected ***n *= 548	**Diagnosed with cancer ***n *= 192
**Clinical characteristics**		
Age at venipuncture (years)	61 (58, 64)	61 (58, 64)
Total PSA (ng/ml)	4.21 (3.39, 5.55)	5.81 (4.07, 10.4)
Free PSA (ng/ml)	0.87 (0.66, 1.25)	0.83 (0.60, 1.35)
Intact PSA (ng/ml)	0.37 (0.28, 0.52)	0.43 (0.29, 0.73)
Human kallikrein 2 (ng/ml)	0.046 (0.030, 0.068)	0.076 (0.050, 0.128)

**Tumor characteristics**		
Abnormal DRE	50 (9%)	76 (40%)
Biopsy Gleason score		
≤6		152 (79%)
7		33 (17%)
≥8		7 (4%)

Data are median (interquartile range) or frequency (percentage). DRE, digital rectal examination; PSA, prostate specific antigen.

**Table 2:** Number of cancers by prostate specific antigen (PSA) level

**Total PSA (ng/ml)**	**No cancer detected**	**Low-grade cancer**	**High-grade cancer**
2.6 to 4.0	241 (84%)	43 (15%)	3 (1%)
4.1 to 10.0	274 (74%)	79 (21%)	18 (5%)
10.1 to 20.0	27 (50%)	18 (33%)	9 (17%)
>20.0	6 (21%)	12 (43%)	10 (36%)

Cancers with biopsy Gleason score 7 and higher were considered high grade.

### Prediction models

**Figure 1:**
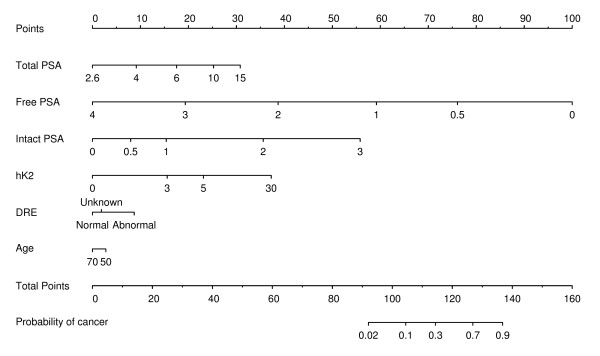
**Nomogram to calculate the risk of prostate cancer on biopsy**. All markers are given in nanograms per milliliter, except for human kallikrein 2, which is given in 10 pg/ml. The model can be used for a man with elevated prostate specific antigen (PSA) at his first PSA test. tPSA, total PSA; fPSA, free PSA; iPSA, intact PSA.

Results of multivariable analysis are shown in Table 3. Our first analysis considered only those predictors obtained from the laboratory. The AUC of the laboratory base model (age+total PSA) was 0.68. Addition of all markers enhanced the AUC of the laboratory base model to 0.83. Predictive accuracy of the full model was higher than that of the base model (*p *< 0.0005); because free PSA is widely used in clinical practice, we also compared the AUC of the full model to that of the base model plus free PSA (*p *= 0.005). Our second analysis considered an additional clinical predictor, the DRE result. The AUC of the clinical base model (age+total PSA+digital rectal exam) was 0.72. As was the case for the laboratory model, all markers enhanced the AUC of the clinical base model; the AUC of the clinical full model was 0.84 (*p *< 0.0005 compared with the clinical base model and *p *= 0.025 compared with clinical base model plus free PSA). The full model incorporating all four kallikrein markers is shown as a nomogram in Figure 1. Note that despite its moderate impact when added to the base model, intact PSA is a statistically significant marker in the full model: this appears to be because it is the ratio between intact and free PSA that is informative, and thus intact PSA is only of value when free PSA is included in the model.

**Table 3:** Predictive accuracy (area-under-the-curve) of kallikreins, with correction by repeated 10-fold cross-validation

**Predictor**	**Laboratory Model**		**Clinical Model**	
	**Any cancer**	**High-grade cancer**	**Any cancer**	**High-grade cancer**

Base model	0.680 (0.636, 0.727)	0.816 (0.741, 0.881)	0.724 (0.677, 0.771)	0.868 (0.795, 0.925)
Base model+Free PSA	0.762 (0.727, 0.807)	0.832 (0.780, 0.916)	0.779 (0.746, 0.828)	0.867 (0.830, 0.941)
Base model+Intact PSA	0.688 (0.651, 0.740)	0.798 (0.760, 0.892)	0.724 (0.687, 0.774)	0.853 (0.822, 0.930)
Base model+hK2	0.725 (0.686, 0.772)	0.826 (0.771, 0.902)	0.760 (0.725, 0.809)	0.864 (0.803, 0.933)

Full model	0.832 (0.804, 0.874)	0.870 (0.841, 0.937)	0.836 (0.810, 0.880)	0.903 (0.860, 0.960)

The base model for the laboratory model includes age and total prostate specific antigen (PSA), and for the clinical model includes age, total PSA, and digital rectal examination result. The full model includes the base model plus free PSA, intact PSA, and human kallikrein 2 (hK2). Cancers with biopsy Gleason score 7 and higher were considered high grade. 95% confidence intervals are given in parentheses, and were calculated using bootstrap methods with 1000 replications.

We performed additional analyses for the outcome of high-grade cancer (Table 3): each of the four kallikrein forms individually, and the full model including the four kallikrein panel, are associated with higher AUC than models incorporating PSA alone. AUCs were increased from 0.82 to 0.87 for the laboratory model and from 0.87 to 0.90 for the clinical model. However, there were only 40 high-grade cancers, and the confidence intervals for the AUCs are consequently very wide. The p values for the hypothesis that addition of free PSA, intact PSA and hK2 improved predictive accuracy (that is, increased the AUC) for detecting high-grade cancer were 0.04 and 0.16 for the laboratory and clinical models, respectively. Given the low number of events, and the possibly questionable properties of cross-validation with highly overfit models, we repeated our analyses without non-linear terms. Although predictive accuracy was lower for all models, the improvement in predictive accuracy associated with the kallikrein panel was similar: AUC increased from 0.78 to 0.86 and from 0.85 to 0.87 for the laboratory and clinical models, respectively.

### Decision curve analysis

**Figure 2:**
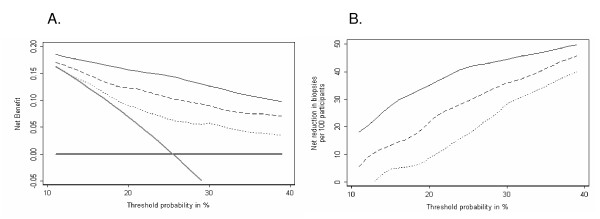
**Decision curve analysis, laboratory model**. The dotted line is for base model (age and total prostate specific antigen (PSA)); the dashed line is for base model plus free PSA; the thin black solid line is for full model (age, total PSA, free PSA, intact PSA, and human kallikrein 2). As a comparison, the thin gray line is for the strategy of biopsying all men and the thick black line for biopsying no men. (a) The net benefit, interpreted as the number of cases of prostate cancer identified per patient keeping the rate of unnecessary biopsy constant. (b) The reduction in the rate of unnecessary biopsy keeping number of cases of prostate cancer identified constant.

**Figure 3:**
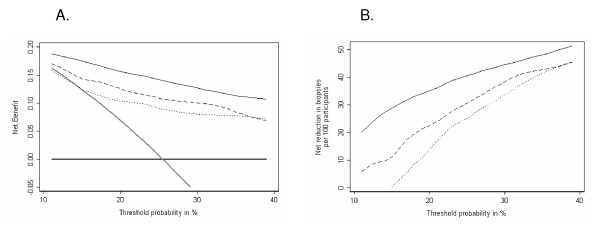
**Decision curve analysis, clinical model**. The dotted line is for base model (age, digital rectal examination (DRE) and total prostate specific antigen (PSA)); the dashed line is for base model plus free PSA; the thin black solid line is for full model (age, DRE, total PSA, free PSA, intact PSA, and human kallikrein 2). As comparison, the thin gray line is for the strategy of biopsying all men and the thick black line for biopsying no men. (a) The net benefit, interpreted as the number of cases of prostate cancer identified per patient keeping the rate of unnecessary biopsy constant. (b) The reduction in the rate of unnecessary biopsy keeping number of cases of prostate cancer identified constant.

The results of the decision curve analysis are shown in Figure 2 for the laboratory model and Figure 3 for the clinical model. For both, use of a model based on all four kallikrein forms has higher net benefit, that is, would lead to superior clinical results, than the strategy of either biopsying all or no men, at all plausible threshold probabilities for biopsy. It is also superior to the base model and to the base model plus free PSA. Figures 2(b) and 3(b) illustrate the extent of clinical benefit achieved by using the models incorporating kallikreins in terms of reduction in biopsy rates. For example, at a threshold probability of 20%, basing biopsy decisions on the full kallikrein laboratory model is equivalent to a strategy that reduced the number of biopsies by 35% but which missed no cancers. To illustrate these findings further, Table 4 shows the results of a strategy of biopsying men with a 20% or greater risk of prostate cancer. Biopsying on the basis of the full laboratory model would spare 424 (57%) men from biopsy and miss only 31 out of 152 low-grade and 3 of 40 high-grade cancers.

**Table 4:** Reduction in biopsies/cancers detected, as compared with the current study, using as a threshold for biopsy a 20% or higher probability of cancer

	**Number of biopsies**		**Number of cancers**		**Number of high-grade cancers**	
	
	**Performed**	**Reduced (%)**	**Caught**	**Missed (%)**	**Caught**	**Missed (%)**
**Biopsy all**	740	n/a	192	n/a	40	n/a
						
**Laboratory model**						
Base	489	251 (34%)	153	39 (20%)	38	2 (5%)
Base+free PSA	380	360 (49%)	152	40 (21%)	36	4 (10%)
Full	316	424 (57%)	161	31 (16%)	37	3 (8%)
						
**Clinical model**						
Base	344	396 (54%)	136	56 (29%)	37	3 (8%)
Base+free PSA	336	404 (55%)	147	45 (23%)	38	2 (5%)
Full	297	443 (60%)	159	33 (17%)	39	1 (3%)

The base model for the laboratory model includes age and total prostate specific antigen (PSA), and for the clinical model includes age, total PSA, and digital rectal examination result. The full model includes the base model plus free PSA, intact PSA, and human kallikrein 2. Cancers with biopsy Gleason score 7 and higher were considered high grade. Percentages given in parentheses represent the percentage as compared with the current study.

### Sensitivity analysis

Our analysis was based on measurement of fresh samples for total and free PSA but frozen, stored and thawed samples for intact PSA and hK2. As a sensitivity analysis, we repeated all analyses using total and free PSA measurements made on frozen and thawed samples. For the laboratory model, the AUC for the base model was 0.66, for base plus free PSA was 0.70, and for the full model was 0.77. The corresponding figures for the clinical model were 0.71, 0.72, and 0.79, respectively. Although the predictive accuracy of total and free PSA is clearly affected by freezing and thawing, our principal finding, that a four-marker panel has importantly greater predictive accuracy than a base model including total PSA alone, is unaffected. As a second sensitivity analysis, we looked at our model for probability thresholds other than our pre-specified 10% to 40% range: the net benefit of the full panel was superior to all alternatives for thresholds above 5% and less than 75%. It is difficult to believe that a clinician would undertake 19 biopsies to find a single cancer (the 5% threshold) or that any patient would believe an unnecessary biopsy to be three times worse than a missed cancer (the 75% threshold).

Of the 956 men with an elevated PSA during round 1, 858 (90%) presented for biopsy. Median total PSA was higher in biopsied men (4.41 versus 3.16 ng/ml in those not biopsied). Given these low levels of missing data, and moderate differences between groups, we did not expect any important levels of verification bias. Indeed, none of the AUCs we examined were adjusted by more than 0.005 by verification bias correction, confirming that verification bias has a negligible impact on our findings.

Patients in this cohort received sextant biopsy. Currently many centers use more extended sampling, such as 12 cores. To examine the effect of biopsy scheme, we examined the subsequent biopsy histories of patients who had negative biopsies in the first round. Fifty-six of these patients had a positive biopsy during the subsequent two screening rounds (that is, within 4 years). This constitutes a detection rate 30% higher than when analyzing first round biopsy only, broadly comparable to what has been reported for direct comparisons between sextant and more extended biopsy [38-40]. We then repeated our analyses assuming that all of these patients would have had cancer detected in the first round, had they received a biopsy with 12 or more cores. Although the predictive accuracy of all models was slightly reduced, the increment in predictive accuracy from the kallikrein panel was very similar (laboratory model: base 0.654 versus full 0.793; clinical model: base 0.693 versus full 0.800) suggesting that our results are robust to type of biopsy.

## Discussion

In this study, we analyzed kallikreins in bloods drawn from men in a randomized trial of prostate screening. We found that, in previously unscreened men with elevated PSA, a statistical model including four markers (total PSA, free PSA, intact PSA and hK2) could predict the result of biopsy more accurately than a model incorporating total PSA and age alone. Moreover, we were able to show using decision analytic methods that application of the model would lead to notably superior clinical outcomes than the current strategy of biopsying all men with elevated PSA.

PSA forms and hK2 have previously been examined to determine their association with biopsy outcome in men with elevated PSA. Although most studies have found that these markers are of value [7,10,14-17], findings have not always been consistent and several authors have questioned the clinical value of the most widely investigated marker, free PSA [6]. This may be because previous studies have studied referral populations, where PSA screening history was undefined and, as pointed out in the introduction, the characteristics of screening tests may vary depending on the number of screens. Moreover, few prior studies have used appropriate decision analytic methods: analyses that focus on the 'diagnostic gray zone' (for example, PSA of 4 to 10 ng/ml) [6,41], or those that compare the specificity of PSA versus PSA plus free PSA at a fixed sensitivity (for example, 90%) [42,43] have a questionable theoretical bias because they use criteria chosen without clear reference to the relative harms of unnecessary biopsy compared with a missed cancer. That said, many studies do not use a decision analytic perspective at all and report only odds ratios and *p*-values.

With respect specifically to free PSA, the majority of previous studies have used frozen and thawed samples [6]. We have found that storage and repeated freeze-thaw cycles lower the value of free PSA: the increment in predictive accuracy associated with free PSA was 0.082 when measured fresh compared with only 0.042 for repeatedly frozen and thawed free PSA. The degradation of intact PSA associated with freezing, storage and thawing appears similar to that of free PSA [17]. It is thus possible that the value of the four-marker panel could be even higher if all four markers were measured on fresh samples, as would be the case in routine clinical practice.

We have conducted preliminary studies that could be subject to the criticisms of the prior literature outlined above. For example, we examined the association between cancer and multiple markers, including the kallikreins evaluated here as well as urokinase forms, in a referral cohort, focusing on patients with PSA of 4 to 10 ng/ml and a negative DRE [44]. Although our results are broadly comparable, with the addition of free and nicked PSA to a base model of total PSA and age leading to increases in AUC, the marginal benefit is greater in the current analyses. This may be explained by the use of frozen and re-thawed serum samples in the previous study and possibly by the greater heterogeneity in age and total PSA in the referral cohort.

Our results for a model including PSA and age alone can be compared to the 'risk calculator' developed by Thompson et al. using data from the Prostate Cancer Prevention Trial (PCPT) [45]. The AUCs for any cancer and high-grade cancer were 0.681 and 0.781, similar to our findings for PSA alone of 0.680 and 0.816. Although there are important differences between the PCPT and ERSPC populations, we believe that these are countervailing. For example, ERSPC patients were younger and not previously screened, both factors which would tend to increase the predictive value of PSA; however, only men with elevated PSAs were biopsied, reducing heterogeneity of PSA levels and thus its predictive value. Nonetheless, the relative closeness of our results to those of the PCPT does suggest the applicability of our findings to a US population.

Of the four markers we examined, assays for total and free PSA are currently widely available. Assays for intact PSA and hK2 require greater sophistication [46,47], but are based on similar principles to the total and free PSA assays. The four assays could therefore be readily commercialized into a single test at relatively low cost. The only cost additional to ascertaining total PSA would be that of purchasing reagents. A commercial enzyme-linked immunosorbent assay (ELISA) kit for 'rare' tumor markers, such as chromogranin A or osteoprotogerin, ranges usually between $500 and $800 for 96 tests. We therefore estimate that the panel of markers would cost only $30 to $40 more than testing total PSA alone. Were our findings to be replicated, they would have immediate practical application for men with elevated PSA.

A limitation of our study concerns its applicability to men who have had previous PSA tests. The prevalence of prior PSA tests in our sample is very low, and the characteristics of screening tests often change with repeat screens. That said, while it is possible that the overall accuracy of our full model may decrease, it seems likely that the accuracy of the model including only age and total PSA would also decrease. If so, the improvement in accuracy associated with the additional markers would not significantly change. Nonetheless, we intend to validate our models in men who have undergone a prior PSA test. We also plan to evaluate our models in other ERSPC cohorts.

In summary, we have found that adding information on kallikreins other than PSA can help predict the result of biopsy in men with elevated PSA. Our models can therefore be used to determine which men should be advised to have biopsy and which might be advised to continue screening, but defer biopsy until there was stronger evidence of malignancy.

## Competing interests

HL holds patents for free PSA and hK2 assays and, with KP, is named as co-inventor on a patent application for intact/nicked PSA-assays.

## Authors' contributions

Each author of this research paper has directly participated in planning, execution, or analysis of the study, and they have read and approved the final submitted version. AJV and HL were key investigators and responsible for study design, data analyses, and the final version of the manuscript. AMC and AJV were responsible for all biostatistical analyses and workup. JH is Principal Investigator for the ERSPC study in Sweden and together with GA was responsible for enrolling ERSPC study participants and performing biopsies on eligible participants. C–GP was responsible for histopathological assessments of all prostate biopsies cores. HL and CB were responsible for measuring all the PSA forms and hK2. KP was responsible for production, standardization, and quality control of assay reagents to measure hK2 and intact PSA. PTS actively contributed to all elements of the study. AJV had full access to all of the data in the study and takes responsibility for the accuracy of the data analysis. HL was responsible for the laboratory studies and takes responsibility for the accuracy of the data. All authors have read and approved the final manuscript.

## Pre-publication history

The pre-publication history for this paper can be accessed here:


